# Biosynthesis of Silver Nanoparticles Using *Aegle marmelos* (Bael) Fruit Extract and Its Application to Prevent Adhesion of Bacteria: A Strategy to Control Microfouling

**DOI:** 10.1155/2014/949538

**Published:** 2014-09-02

**Authors:** A. Nithya Deva Krupa, Vimala Raghavan

**Affiliations:** School of Biosciences and Technology, VIT University, Vellore, Tamil Nadu 632014, India

## Abstract

Marine biofilms formed due to adhesion of bacteria and other microorganisms on submerged surfaces are generally considered to be a major form of microfouling. Subsequent attachment of larvae of higher organisms like barnacles, mussels, and so forth, on marine biofilms, causes macrofouling. Several approaches have been used to prevent micro- and macrofouling. Silver nanoparticles (AgNPs) are known to exhibit strong inhibitory and antimicrobial activity. Biological synthesis of AgNPs is rapidly gaining importance due to its growing success. Hence, the present study is focused on the biosynthesis of AgNPs using fruit extract of *Aegle marmelos* and its characterization through UV-Vis spectrophotometer, X-ray diffractometer (XRD), Fourier transform infrared spectroscopy (FTIR), and atomic force microscopy (AFM). Further isolation and identification of marine biofilm forming bacteria were carried out through 16S rDNA analysis. The antimicrofouling effect of the biosynthesized AgNPs was tested against marine biofilm forming bacteria and the results suggested that it could effectively inhibit biofilm formation. This preliminary study has proved that AgNPs may be used as antimicrofouling agent for the prevention of biofouling in the early stages.

## 1. Introduction

Marine biofouling is one of the major problems encountered on the man-made objects in the marine environment. Biofouling has been defined as the undesirable accumulation of microorganisms, plants, and animals on artificial surfaces immersed in a common matrix [1]. The establishment of fouling community takes place in several stages. Initially, any submerged surface gets coated by a conditioning film consisting of organic and inorganic molecules. The subsequent onset of macrofouling may be preceded by the formation of bacterial biofilms (bacterial fouling) and such a biofilm may have a deleterious effect on the ability of the surface to remain free from larger fouling organisms. Use of chemical antifouling agents is one of the common and easy approaches to control fouling caused by micro- and macrofoulers [2]. However, many antimicrobial materials are less effective on microorganisms in biofilms compared to their planktonic counterparts [3]. Therefore, high concentrations of chemical antifoulants are required for the effective control of fouling resulting in harmful secondary effects. Future research on the antifouling strategies may target the formation of the conditioning layer as a way to prevent subsequent colonization of the surface [4].

In the recent past, nanoparticles are gaining importance in the fields of biology, medicine, and electronics owing to their unique physical and biological properties. Silver has long been known to have strong inhibitory and bactericidal effects as well as a broad spectrum of antimicrobial activities, even at low concentrations [5]. Hence, among the metal nanoparticles, AgNPs have received much attention in various fields including biomedical device coating [6], water purification [7], and food packages [8]. Furthermore, it is considered to be one of the less toxic and safe antibacterial agents to higher animals [9]. AgNPs have been examined for their ability to reduce microbial infections in skin [10] and burn wounds [11] and also to prevent bacterial colonization on various surface devices such as catheters [12]. There are several physical and chemical methods employed for the synthesis of AgNPs [13], but these methods have created great environmental concerns including the use of toxic compounds and generation of hazardous by-products. On the other hand, the use of biological entities, namely, microorganisms and plants, has been explored for the synthesis of AgNPs and has been found to be a clean, nontoxic, and environmentally acceptable method. The use of microorganism in the synthesis of AgNPs has some of the disadvantages which include maintaining cell culture in aseptic environment and prolonged incubation period. However, plant mediated synthesis of AgNPs offers several advantages such as cost effectiveness and nontoxic and eco-friendly products [14]. Plant extracts act as reducing and capping agents for the synthesis of AgNPs and the particles formed are more stable with various shapes and sizes, making it a more efficient method than chemical and microbial synthesis. Many types of plants have been employed for the synthesis of AgNPs, namely,* Vitex negundo* [13],* Plectranthus amboinicus* [15],* Gardenia jasminoides* [16],* Origanum vulgare* [17], and fruit extract of* Syzygium cumini*.

*Aegle marmelos* is a species of tree native to India and it belongs to the family Rutaceae. It is commonly known as bael, golden apple, and Bengal quince which is seen throughout southeast Asia. The fragrant leaves and fruit of the plant have medicinal value and were used to treat dyspepsia and sinusitis. Leaves of the* A. marmelos* contain alkaloids of which aegeline (N-[2-hydroxy-2(4-methoxyphenyl)ethyl]-3-phenyl-2-propenamide) is a known constituent and is consumed as a dietary supplement. Fruits of the plant contain several bioactive compounds which include marmelosin, luvangetin, auraptene, psoralen, marmelide, and tannin. In the present study, we have demonstrated the prospect of using* Aegle marmelos* fruit extracts for the synthesis of AgNPs. Further, the antimicrofouling activity of the biosynthesized AgNPs was tested against marine biofilm forming bacteria which were isolated from ship's hull and identified through 16S rDNA analysis.

## 2. Materials and Methods

### 2.1. Media and Chemicals

All the media components were purchased from Hi-Media Laboratories Pvt Ltd., Mumbai, India, and silver nitrate was purchased from Sigma Aldrich Chemicals, India. The healthy fruit of* A. marmelos* was collected from a temple in Vellore, Tamil Nadu, India.

### 2.2. Preparation of Fruit Extract

In order to prepare the aqueous fruit extract, 50 g of fresh* A. marmelos* fruit was surface-cleaned using tap water and then double-distilled water. The fruit was dried in an oven at 60°C for 48 h and then it was crushed into powder using mortar and pestle. The aqueous extract was prepared by heating the ground powder in 100 mL of Milli-Q water at 60°C for 10–15 min in a water bath and the resulting extract was filtered using Whatman number 1 filter paper. The filtrate was used for the synthesis of AgNPs.

### 2.3. Synthesis of Silver Nanoparticles Using* A. marmelos* Fruit Extract

Biosynthesis of AgNPs was carried out by the simple reduction of silver nitrate using aqueous fruit extract of* A. marmelos* by following the standard published literature with minor modifications. In a typical procedure, 12 mL of the prepared fruit extract was added to 88 mL of 1 mM silver nitrate solution and it was incubated in dark at room temperature. Then, the formation of nanoparticles was confirmed by visual color change from yellow to dark brown.

### 2.4. Characterization

Green synthesized AgNPs were first characterized by UV-Vis spectrophotometer (Perkin-Elmer, Germany) in the range 300–800 nm at a resolution of 1 nm. The synthesized AgNPs were purified and freeze-dried using a lyophilizer and the powered sample was analysed using X-ray diffractometer. The XRD patterns were collected on Bruker AXS D8 Advance X-ray diffractometer with Cu K*α* radiation of wavelength 1.541° and scanning angle 2*θ* over the range of 10°–80°. Further characterization of the synthesized nanoparticles involved Fourier transform infrared spectroscopy (Perkin-Elmer, Germany), using the lyophilized sample by KBr pellet technique in the range of 400–4000 cm^−1^. The size and morphological characterization of the synthesized nanoparticles were studied using atomic force microscopy (Nanosurf ARITIDIS). The microscopic images were recorded with silicon cantilever with force constant 0.22–0.77 N/m and tip height 10–12 nm in the contact mode.

### 2.5. Sample Collection and Isolation of Marine Biofilm Forming Bacteria from Ship's Hull

Biofilm samples were collected from the hull of the ships anchored at Royapuram Harbor (13°6′15′′N 80°17′30′′E) Chennai, Tamil Nadu, India. The collected samples were brought to the laboratory in a sterile container and stored in refrigerator at 4°C. Isolation of bacteria was carried out by serial dilution of the collected samples and plating over Zobell Marine Agar (ZMA) within 48 h from the time of sampling. The inoculated plates were incubated at 37°C for 24–48 h for the growth of the colonies; morphologically distinct colonies were purified and screened for their biofilm forming ability as described by O'Toole and Kolter [18]. Further, the isolates that showed maximum biofilm forming ability/fouling activity were selected and identified through molecular techniques.

### 2.6. Molecular Characterization and Identification

Marine biofilm forming bacteria were characterized and identified by 16S rDNA analysis. Genomic DNA was isolated from the pure cultures and fragments corresponding to 16S rRNA were amplified in a PCR using the following forward (5′-CWG RCC TAN CAC ATG SAA GTC-3′) and reverse (5′-GRC GGW GTG TAC NAG GC-3′) primers (single letter code: R = A or G, S = C or G, W = A or T, N = A or C or G or T, W = A or T). These sequences were compared with the 16S rDNA sequence in NCBI database and nucleotide sequence similarities were determined using BLASTN [19]. The sequences were aligned using multiple sequence alignment software CLUSTALW2 program employing the neighbor joining algorithm to establish the phylogeny [20].

### 2.7. Analysis of Antimicrofouling Effect of Synthesized AgNPs

#### 2.7.1. Well Diffusion Method

The bactericidal effect of the biologically synthesized AgNPs against marine biofilm forming bacteria was assessed by the standard agar well diffusion technique [21]. The test organisms were grown in nutrient broth for 24 h and the overnight broth cultures of each bacterial isolate were adjusted to 0.5 McFarland standards (0.5–2.5 × 10^23^) CFU mL^−1^ and a lawn culture was made on Mueller Hinton agar (MHA) plates. Lyophilized AgNP was dissolved in sterile distilled water and sonicated in order to prevent the agglomeration of particles. Five wells each of 6 mm diameter were made on each plate and the synthesized AgNP solution at a concentration of 20, 40, 60, and 80 *μ*g mL^−1^ was loaded in each well. Wells in the centre of the plates without addition of nanoparticles were maintained as control. The plates were then incubated at 37°C for 24 h and the zone of inhibition was calculated by subtracting the well diameter from total inhibition zone diameter.

#### 2.7.2. Extraction of Extracellular Polysaccharides (EPS)

The effect of AgNPs on the production of EPS was studied by the extraction and quantification of EPS from the marine biofilm forming bacteria. Briefly, the isolates were grown overnight at different concentrations of AgNPs (0, 20, 40, 60, and 80 *μ*g mL^−1^) in nutrient broth at 37°C. Extraction of EPS from the bacterial isolates was carried out according to Kumar et al. [22]. The overnight grown bacterial cultures were centrifuged at 8000 rpm for 10 min at 4°C and the supernatants were collected. Three volumes of 95% ice cold ethanol were added to the supernatants in order to precipitate out the EPS. The crude EPS thus obtained was separated out by centrifugation at 10,000 rpm for 15 min and the weight of the extracted EPS was determined after air drying the samples for 48 h at room temperature.

#### 2.7.3. Antibiofilm Activity

The antibiofilm activity was performed by microtiter plate method as per our earlier report [23]. The bacterial strains were grown overnight in nutrient broth at 37°C with shaking at 150 rpm. 100 *μ*L of bacterial cultures were inoculated in individual wells of sterile 96-well microtiter plate with AgNPs (80 *μ*g), and control wells were maintained without addition of nanoparticles. Then, the plate was incubated at 37°C for 3 days in order to check the ability of the bacterial isolates to form biofilms. After incubation period, the culture in each well was removed carefully and rinsed with sterile phosphate buffered saline (PBS) in order to remove the loosely attached bacterial cells. Biofilms formed by the adherent cells were quantified by staining the wells with 100 *μ*L of crystal violet (0.1% W/V) for 20 min. After staining, the plate was rinsed with tap water and crystal violet was solubilized with 99% ethanol. The optical densities (OD) of stained adherent bacteria were determined with a microtiter plate reader at 570 nm (OD_570_ nm) which directly corresponds to the amount of biofilm.

### 2.8. Statistical Analysis

All experiments were carried out in triplicate and the results were interpreted using GraphPad Prism 6 statistical software. The statistical analysis of experimental data utilized two-way ANOVA and statistical significance was accepted when a *P* value was less than 0.05

## 3. Results and Discussion

### 3.1. Characterization of AgNPs

#### 3.1.1. UV-Visible Spectroscopic Analysis

**Figure 1:**
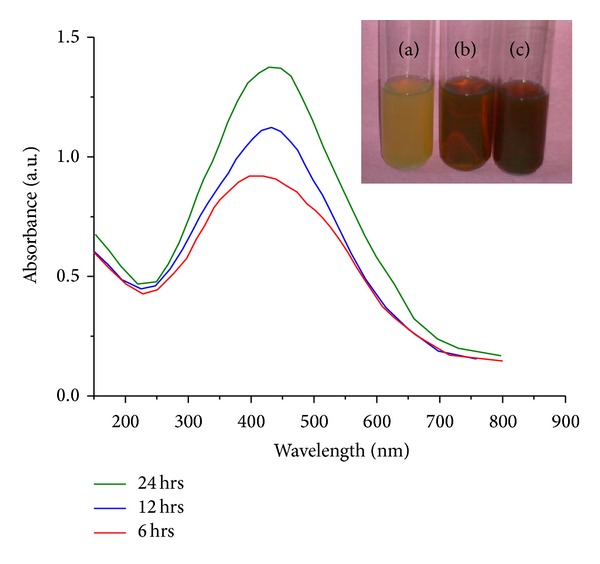
UV-Vis spectrum of AgNPs synthesized using fruit extract of* A. marmelos*: (a) control; (b) after 12 h; (c) after 24 h.

In order to confirm the formation of AgNPs, the aqueous fruit extract of* A. marmelos* treated with 1 mM AgNO_3_ solution was monitored by UV-Vis absorption spectrum in the range of 200–800 nm. An absorption peak centered at 423 nm characteristic of AgNPs was observed (Figure 1). Surface plasmon resonance (SPR) patterns are characteristic of metal particles which are associated with the particle size, stabilizing molecules, surface adsorbed particles, and dielectric constant of the medium. A single SPR band corresponds to the spherical nanoparticles, whereas two or more SPR bands correspond to the anisotropic molecules [24]. In the present study, a single SPR band exhibited by the reaction mixture reveals the spherical shape of the AgNPs. The intensity of the SPR peak increased with reaction time indicating the increasing concentration of AgNPs. At 24 h of reaction time, the absorbance reached its maximum after which there is no change in the position of the peak indicating the end of the reaction.

#### 3.1.2. XRD

**Figure 2:**
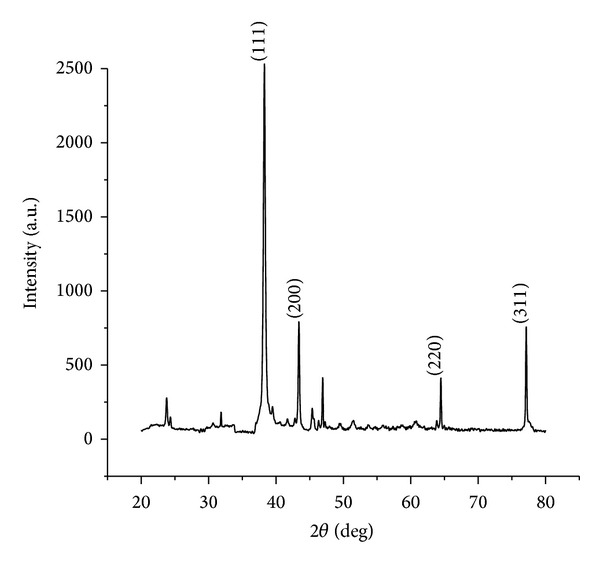
X-ray diffraction pattern of synthesized AgNPs.

The crystalline nature of the green synthesized AgNPs by the fruit extract* A. marmelos* was confirmed by X-ray diffraction studies (Figure 2). Four distinct peaks at 38.26°, 43.38°, 64.4°, and 77.05° indicated the (111), (200), (220), and (311) reflections of metallic silver. XRD pattern also represents the face-centered cubic structure of silver which closely matched the reported reference values of Joint Committee on Power Diffraction Standards (JCPDS) 03-0921. The sharp peaks clearly confirm the crystalline nature of the synthesized nanoparticles which is in good agreement with the earlier reports [25].

#### 3.1.3. FTIR

**Figure 3:**
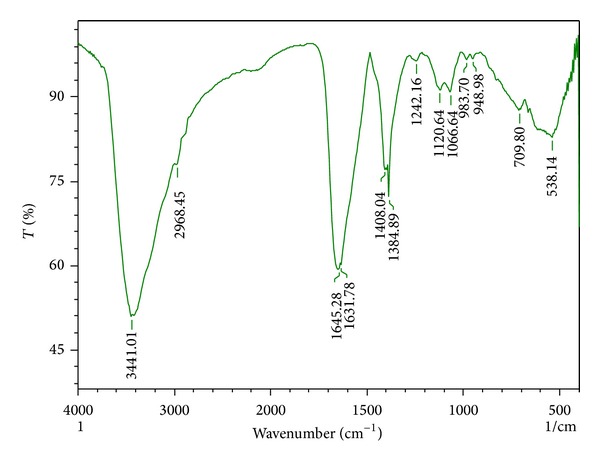
FTIR spectrum of fruit extract of* A. marmelos* treated with 1 mM AgNO_3_.

FTIR spectrum of the synthesized AgNPs is shown in Figure 3 which reveals the possible biomolecules present in the fruit extract which is accountable for the reduction of silver ions and its interaction with the AgNPs. The IR spectrum shows intense bands at 3441.01 cm^−1^, 1645.28 cm^−1^, 1631.78 cm^−1^, and 1409.04 cm^−1^. The broad band at 3441.01 cm^−1^ corresponds to the strong stretching vibrations of hydroxyl group (–OH) of phenolic compounds [26]. The sharp intense peaks at 1645.28 cm^−1^ and 1631.78 cm^−1^ can be attributed to the –C=O– and –C=C– stretching vibrations, which indicates the presence of flavonoids and terpenoids in the fruit extract of* A. marmelos*. The two peaks at 1120.64 cm^−1^ and 1066.64 cm^−1^ can be attributed to the –C–O– stretching vibrations of carboxylic acid, ester, and ether groups of the proteins and metabolites present in the extract that may be involved in the reduction process [27]. The band at 1408.04 cm^−1^ corresponds to the C=N stretching vibration of aromatic amines. Weak signals observed at 700.12 cm^−1^, 538.04 cm^−1^, and 983.70 cm^−1^ correspond to –C–Cl– and –C–OCH_3_ stretching modes of the alkyl halides and alkenes, respectively [28]. Thus, from the IR spectrum, it may be assumed that these biomolecules have major role in the bioreduction as well as in the stabilization of AgNPs.

#### 3.1.4. AFM

**Figure 4:**
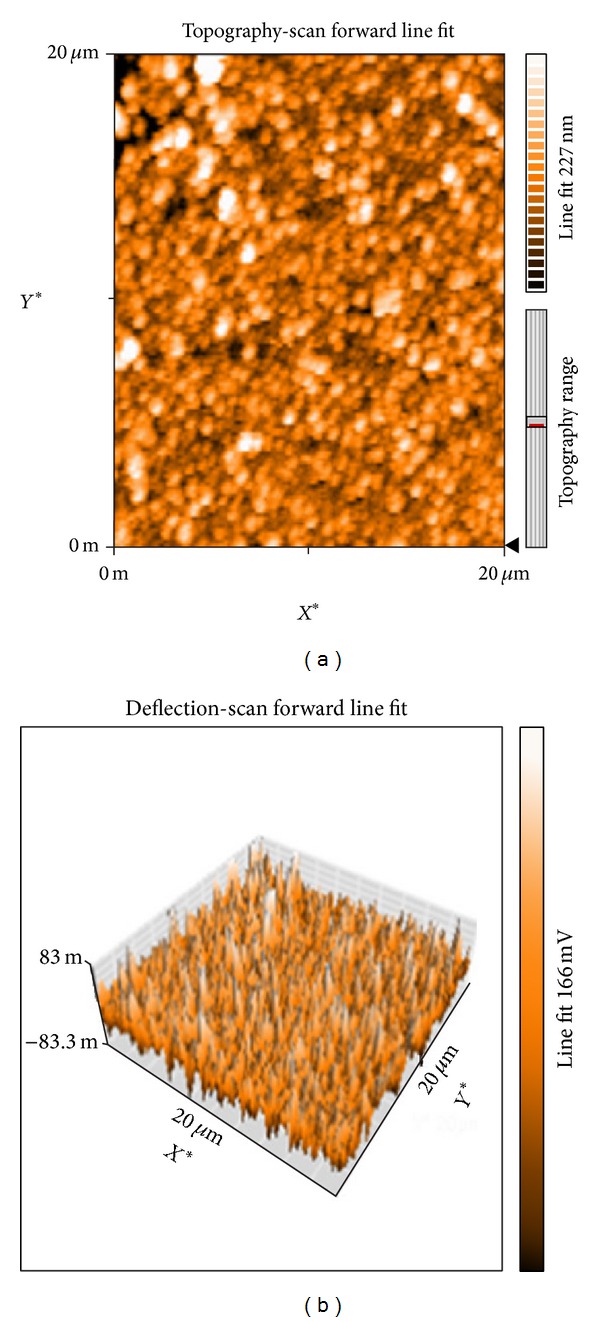
Atomic force microscopic images of the synthesized AgNPs: (a) aerial view showing topographical characteristics; (b) 3D view.

The biosynthesized AgNPs were further confirmed by AFM micrographic images. The images were obtained using silicon cantilevers with force constant 0.02–0.77 N/m in a contact mode [29]. The lyophilized sample was dissolved in acetone and spin-coated using apex instrument spin coater and then it was dried for 15 min prior to the analysis. The particles were spherical in shape and the size of individual particles was found to be 34.7 nm. The topographical image of the synthesized nanoparticles shows the individual particles as well as agglomeration (Figure 4).

### 3.2. Isolation and Molecular Identification

Totally, 12 different bacterial strains were isolated and screened for their ability to form biofilms. Among the 12 isolates, five isolates which showed maximum fouling activity were selected and, after 16S rDNA sequencing, they were identified as* Pseudomonas otitidis* strain NV1,* Pseudomonas aeruginosa* strain NV2,* Enterobacter cloacae* strain NV3,* Microbacterium* sp. NV4, and* Staphylococcus hominis* strain NV5. Their sequences were deposited in GenBank (NCBI) under the accession numbers KF574079, KF574080, KF574081, KF574082, and KF574083, respectively.

### 3.3. Antimicrofouling Studies

#### 3.3.1. Well Diffusion Assay

The benign synthesis of nanoparticles has led to this novel study where aqueous fruit extract of* A. marmelos* was used in the synthesis of AgNPs and the antimicrofouling effect of the green synthesized AgNPs was tested against the marine biofilm forming bacteria. The AgNPs showed remarkable antibacterial activity against all the five biofilm forming bacteria (*Pseudomonas otitidis* strain NV1,* Pseudomonas aeruginosa* strain NV2,* Enterobacter cloacae* strain NV3,* Microbacterium* sp. NV4, and* Staphylococcus hominis* strain NV5) which was confirmed by the circular zone of inhibition around the well. The zone of inhibition was found to increase with the increase in concentration of AgNPs and the effect was found to be the highest at 80 *μ*g mL^−1^ concentrations (ANOVA, *P* < 0.05). Among the tested isolates, the zone of inhibition was found to be the highest against* Pseudomonas aeruginosa* strain NV2 (12 mm) and* Microbacterium* sp. NV4 (10 mm) and the lowest against* Enterobacter cloacae* strain NV3 (6 mm). In case of* P*.* otitidis* strain NV1 and* S. hominis* strain NV5 it was found to be 9 mm and 7 mm, respectively (Table 1).

#### 3.3.2. Effect of AgNPs on Production of Extracellular Polysaccharides

**Figure 5:**
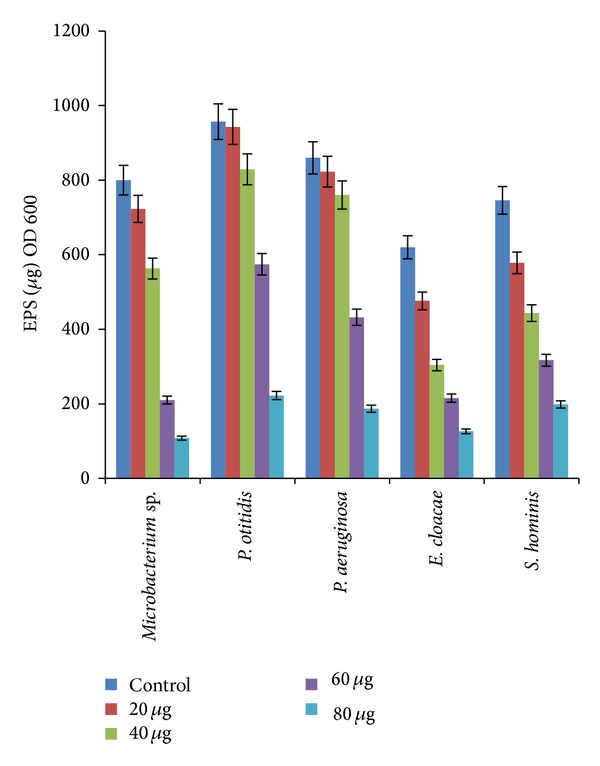
The effect of biosynthesized AgNPs on EPS production by marine biofilm forming bacteria.

**Figure 6:**
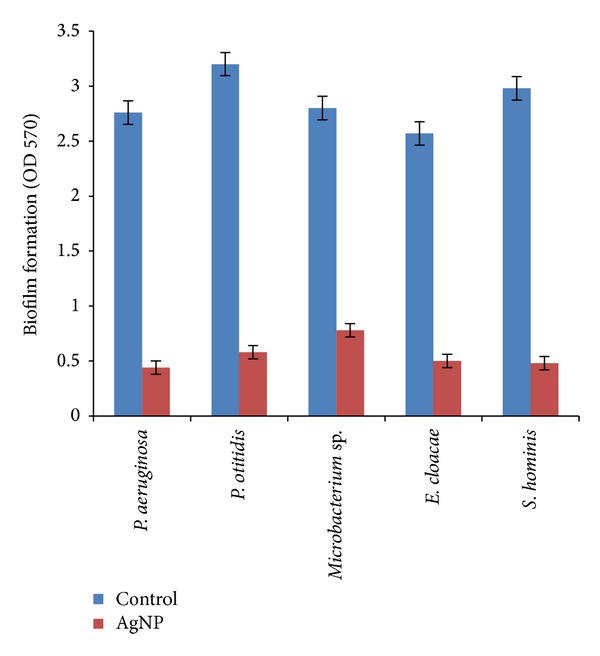
Antibiofilm activity of AgNPs on marine biofilm forming bacteria.

Extracellular polysaccharides are the wide group of secreted polymers that can be highly attached to the cell surface or released as extracellular slime in the surrounding environment of the cell. Biofilm formation in bacteria is closely linked to the production of EPS which acts as the binding agent in the adhesion of bacteria to the submerged surface. In the present study, the effect of AgNPs on the production of EPS by the marine biofilm forming bacteria was determined by the biomass yield (EPS/unit biomass).  Figure 5 shows the dry weight of EPS extracted from the biofilm forming bacteria which can be correlated with the biofilm formation as shown in Figure 6. Among the five biofilm forming bacteria,* Pseudomonas otitidis* strain NV1 was found to produce the maximum amount of EPS, whereas* E. cloacae* strain NV3 produced the least amount. Further, the effect of AgNPs (0, 20, 40, 60, and 80 *μ*g mL^−1^) on the production of EPS was studied and the results are shown in Figure 6. Increase of AgNPs concentration negatively regulated the EPS production (ANOVA, *P* < 0.05). This shows that biosynthesized AgNPs have the ability to block the EPS production, otherwise the biofilm by the bacterial isolates. Kalishwaralal et al. [30] studied the antibiofilm activity of the AgNPs against the biofilms developed by* Pseudomonas aeruginosa* and* Staphylococcus epidermidis*. They demonstrated that nanoparticles could effectively block the synthesis of EPS. According to the literature, bacteria with EPS producing ability may have an advantage in successfully colonizing the surfaces by becoming primary colonizers. It has been reported that the differences in biofilm forming abilities among the bacterial strains could be due to the differences in their innate ability to attach and proliferate on surfaces [31].

#### 3.3.3. Antibiofilm Activity

Further, the antibiofilm activity of the AgNPs was assayed by microtiter plate method which directly quantifies the attached bacteria. The biofilm was detected by staining with crystal violet and by measuring the optical density at 570 nm. The OD values of the control wells were greater than those of the wells loaded with AgNPs (80 *μ*g mL^−1^) (ANOVA, *P* < 0.05). This directly reflects the antibiofilm ability or antifouling property of the biogenic AgNPs (Figure 5). Thus, the above results suggest that the AgNPs can act as potential antimicrofouling agent against marine biofilm forming bacteria. Although several studies have reported the bactericidal effect of silver nanoparticles [9, 32], their mechanism of action is not clear till date. Li et al. [33] studied the mechanism of action of AgNPs against* E. coli* and reported that the nanoparticles were able to damage the structure of bacterial cell membrane and depress the activity of some membranous enzymes, which eventually lead to the death of the bacteria. Similarly, Kora and Arunachalam [34] have reported the association of reactive oxygen species (ROS) and cell membrane damage in the antibacterial mechanism of AgNPs against* Pseudomonas aeruginosa.* As a result of Ag^0^ ionization, Ag^+^ ions can also interact with proteins, specifically with reactive thiol groups (cysteines), increasing the damage to the cells [35]. Thus, from the above reports, it is suggestive that AgNPs may act on marine biofilm forming bacteria by inhibiting the respiratory chain enzymes resulting in the formation of ROS which eventually lead to the death of the bacteria. Therefore, the results obtained directly reveal that biologically synthesized AgNPs not only effectively inhibited the growth of the bacteria but also prevented the biofilm formation.

## 4. Conclusion

Green synthesis of AgNPs from the aqueous fruit extract of* A. marmelos* was reported. Marine biofilm forming bacteria which were isolated from the hull of the ship were identified through 16S rDNA analysis based on their ability to cause microfouling. The ability of the green synthesized nanoparticles to control/prevent the biofilm forming bacterial communities (which were considered as primary colonizers) was studied by conducting the antimicrofouling studies. The nanoparticles were found to control the growth and survival of biofilm forming bacteria effectively which was evident from the antimicrofouling studies. Further work needs to be carried out to prove the field applicability of the synthesized nanoparticles in marine environment. Thus, the present work gives scope for the possible development of formulations containing AgNPs as effective antifouling agent that could prevent microfouling, thereby preventing marine biofouling.

## Figures and Tables

**Table 1:** Antibacterial activity of AgNPs against marine biofilm forming bacteria.

S. number	Organism	Concentration of AgNPs (*µ*g/mL)	Zone of inhibition (mm) (mean of the three replicates)
1	*Pseudomonas aeruginosa* NV2	20	5.0 ± 0.12
		40	7.6 ± 1.23
		60	8.5 ± 0.14
		80	12.0 ± 0.57
		Control	0

2	*Microbacterium *sp. NV4	20	5.2 ± 0.76
		40	8.3 ± 0.54
		60	9.6 ± 1.14
		80	10.7 ± 0.21
		Control	0

3	*Pseudomonas otitidis* NV1	20	4.0 ± 0.67
		40	5.3 ± 0.80
		60	6.1 ± 1.71
		80	9.0 ± 1.20
		Control	0

4	*Staphylococcus hominis* NV5	20	3.0 ± 0.92
		40	4.9 ± 0.60
		60	6.7 ± 0.72
		80	7.2 ± 0.48
		Control	0

5	*Enterobacter cloacae* NV3	20	2.1 ± 0.91
		40	3.2 ± 0.32
		60	5.0 ± 1.30
		80	6.1 ± 1.42
		Control	0
